# Cocaine and MDMA Induce Cellular and Molecular Changes in Adult Neurogenic Systems: Functional Implications

**DOI:** 10.3390/ph4060915

**Published:** 2011-06-17

**Authors:** Vivian Capilla-Gonzalez, Vicente Hernandez-Rabaza

**Affiliations:** 1 Laboratory of Comparative Neurobiology, Instituto Cavanilles de Biodiversidad y Biologia Evolutiva, Universidad de Valencia, Catedratico Jose Beltran 2, 46980, Paterna, Valencia, Spain; 2 Brain Tumor Stem Cell Laboratory, Department of Neurosurgery, Johns Hopkins University School of Medicine, Baltimore, Maryland 21231, USA; 3 Laboratory of Neurobiology, Centro de Investigacion Principe Felipe, Avda Autopista del Saler 16, 46012, Valencia, Spain

**Keywords:** cocaine, MDMA, adult neurogenesis, memory, dentate gyrus

## Abstract

The capacity of the brain to generate new adult neurons is a recent discovery that challenges the old theory of an immutable adult brain. A new and fascinating field of research now focuses on this regenerative process. The two brain systems that constantly produce new adult neurons, known as the adult neurogenic systems, are the dentate gyrus (DG) of the hippocampus and the lateral ventricules/olfactory bulb system. Both systems are involved in memory and learning processes. Different drugs of abuse, such as cocaine and MDMA, have been shown to produce cellular and molecular changes that affect adult neurogenesis. This review summarizes the effects that these drugs have on the adult neurogenic systems. The functional relevance of adult neurogenesis is obscured by the functions of the systems that integrate adult neurons. Therefore, we explore the effects that cocaine and MDMA produce not only on adult neurogenesis, but also on the DG and olfactory bulbs. Finally, we discuss the possible role of new adult neurons in cocaine- and MDMA-induced impairments. We conclude that, although harmful drug effects are produced at multiple physiological and anatomical levels, the specific consequences of reduced hippocampus neurogenesis are unclear and require further exploration.

## 1. Introduction

The adult mammalian brain has two main regions capable of constantly producing new cells: the dentate gyrus (DG) of the hippocampus and the subventricular zone (SVZ) of the lateral ventricles [1-5]. Neural stem cells have been identified as astrocytes in both regions [3,6-8]. In the DG, newly produced cells mature and differentiate into granule neurons, which are incorporated locally into the granular layer [9]. In the SVZ, neuroblasts migrate a long distance via the rostral migratory stream (RMS) to the olfactory bulb (OB), where they differentiate into granular and periglomerular interneurons [10-12]. In both cases, newly differentiated neurons are integrated in specific functional systems [13-16].

The discovery of adult neurogenesis in the second half of the 20^th^ century prompted an explosion of research focused on this process. However, the full relevance of the functional significance of adult neurogenesis is still unclear. It is known that adult neurogenesis ocurrs in all mammalian species, including humans [7,17-19]. Furthermore, the fact that this occurs in the OB and DG neural systems linked to relevant brain functions suggests that this regenerative process has an important functional role. In the 1980s, Nottebohm and colleagues were the first to provide evidence of the possible functional role of neurogenesis in the brains of canaries. They detected proliferative cellular activity in one of brain nuclei that control the birds' song, the high vocal centre, and demonstrated that the newly generated cells were neurons. In addition, they showed an increase in the proliferative rate that corresponded with sing-courtship requirements [20,21].

Recently, different studies have suggested that the production of new neurons in the adult brain is also implicated in functions conferred to the OB and DG [22,23]. The selective ablation of the proliferative population in neurogenic regions by irradiation [24-27] or genetic manipulation [28,29] is known to cause severe cognitive deficits. The data support a relationship between the incorporation of new neurons into the OB and olfactory function [14,15,30]. It is hypothesized that neurogenesis in the OB are involved in both the consolidation of olfactory memory [24,31,32] and the discrimination of odors [33-35]. However, the majority of the research work in this field has focused on adult neurogenesis in the DG. The function of the DG is integrated in the neocortical-hippocampus memory system [36-38], which is involved in the formation of temporal lobe dependent memories, such as episodic memories. Different authors have explored the specific contribution of the DG to the formation of these memories, and the principal theory holds that the DG produces sparse representation in the CA3 auto-association network of the hippocampus system, a process named pattern separation [39]. Currently, adult neurogenesis in the DG has been linked to pattern separation in this region [40]. Other authors have proposed that DG function is related to the formation of some memories, such as working memory, complex spatial learning and associative context memories [41-44], and that new cells may play an important role in these processes [16,29,45-47]. All these results suggest that the key to a deeper knowledge of adult neurogenesis function is a better understanding of DG and OB functions.

Given that adult neurogenesis may be implicated in some brain functions, it is of interest to analyze the factors that may modify neurogenesis. Many studies show that neurogenesis can be modulated by positive and negative factors. For example, exposure to an enriched environment and physical activity increases neurogenesis of granular cells in the DG of adult mice [48-50]. Training animals in hippocampus-dependent tasks also has beneficial effects on adult neurogenesis, which are linked to a higher performance in behavioral tests [51,52]. In contrast, negative factors, such as stress [53] and chemicals-exposures [54,55], lead to a significant decrease in proliferation and viability of neural progenitors. All these results implicate alterations in adult neurogenesis related with disrupted cerebral functions, particularly cognitive deficits and mood disorders [56].

Drugs of abuse, such as nicotine, ethanol, cocaine and amphetamine derivates, have also recently been considered as negative factors that affect adult neurogenesis, mainly in the hippocampus [57-66]. It has been noted that chronic or acute use of these recreational drugs can induce alterations in proliferation rate, survival and differentiation of new cells in neurogenic regions [59,60,67-70]. In addition, some hippocampus-dependent memories are affected by psychomotor drugs in humans and experimental models [70,71], and, as previously mentioned, these memories are influenced by adult hippocampal neurogenesis [22]. The present challenge is to discern the role that new cells play in neurogenic systems in a context as complex as drug-induced deficits. With a focus on this question, we briefly summarize previous data regarding the cellular and molecular changes that cocaine and 3,4-Methylenedioxymethamphetamine (MDMA) cause in the DG and OB system. Afterwards, we will explore the potential effects of both drugs of abuse on adult neurogenesis, and will discuss the possible links between adult neurogenesis and cognitive deficit impairments induced by stimulant drugs.

## 2. Cocaine

Cocaine is one of the most widely consumed illegal drugs in developing countries and is associated with several health problems [72]. This drug acts as an indirect agonist of several neurotransmitters, including dopamine, norepinephrine and serotonin. At the same time, chronic cocaine treatments produce toxicity [73] and an increase in oxidative stress and pro-inflammatory mediators [74]. Furthermore, cocaine exposure induces neuroadaptations in different brain areas [75-77].

### 2.1. Effects of Cocaine on the Dentate Gyrus

In both human users and experimental models some of the cognitive functions linked to the DG are affected by cocaine treatments, such as working and recognition memory [70]. Studies that have explored specific alterations of the DG indicate that cocaine alters neurotransmission at structural and functional levels. The alterations of the polysialylated form of the neural cell adhesion molecule (PSA-NCAM) may modulate synaptic rearrangement and interfere with the synaptic plasticity required for the induction of long term potentiation (LTP) and implicated in long term depression (LTD) [78]. Acute administration of cocaine decreases the numbers of PSA-NCAM-positive cells in the DG, while chronic treatment produces the opposite effect [79]. Furthermore, it has been documented that a single cocaine administration (15 mg/kg) not only affects the number and maturation of the PSA-NCAM-positive cells, but also alters hippocampus LTP generation. Both processes are regulated, in part, by corticoid activity [80]. At the neurotransmitter level, cocaine affects the normal function of different neurotransmitters in the DG. For example, in the serotonergic systems, which are involved in the regulation of anxiety and depression states, these systems are affected by binge administration of cocaine through a down-regulation of serotonin receptors [81]. Acute or chronic administration of cocaine also affects the opioid system in the DG by increasing the production of opioid peptides such as prodynorphin [82]. *In vitro* quantitative receptor autoradiography and *in situ* hybridization studies have revealed a site-specific regulation of receptor expression in the hippocampus. For example, after a chronic administration of cocaine, *N*-methyl-D-aspartate (NMDA) receptor (NR1/NR2A-2C) expression decreases in the DG, but not in other hippocampal subfields, which does not occur with other drugs such as morphine [83]. These modifications can be reversed after the withdrawal period. The amount of the NR1 subunit decreases after chronic administration of cocaine in the nucleus accumbens, globus pallidus and subiculum during the early withdrawal period, while the NR1 mRNA level in the DG significantly increases [84].

On the other hand, in the adult brain, the participation of new hippocampal neurons in some memory processes is firmly established [22,29,85]. The effects of drugs on adult hippocampal neurogenesis seem to induce alterations in memory performance. Several studies have documented that repeated administration of cocaine induces a decrease in the proliferation rate of DG progenitor cells [64,65,86]. However, the acute administration of this drug does not produce changes in the proliferation rate [65,80,87] at high or low doses (0.5 mg/kg versus 1.5 mg/kg) [88]. Furthermore, impairment of cellular division is normalized after a period of withdrawal [89]. Recent studies suggest that chronic cocaine administration induce an increase of the proliferative rate within the DG after a short-term period of abstinence (2-4 days) [90]. In general, however, results seem to indicate that chronic administration of cocaine induces a decrease in the proliferation of DG progenitor cells, and that this effect is normalized after abstinence from the drug. On the other hand, several studies indicate that chronic cocaine treatment, administrated by an experimenter or self-administration, does not affect the survival of new cells [63,65,87,89]. A reduction in the survival of BrdU-positive cells has been published recently using high doses of cocaine self-administration, but not with lower doses [88]. Analyses of immature young neurons reveal that the differentiation of the new cells is not affected by cocaine treatment [65,87]. However, the maturation of new cells can be affected by certain conditions, including long self-administration of cocaine (eight weeks) [89], high doses of drug (1.5 mg/kg) [88], or a combination of withdrawal and specific phenotypic behaviors, such as specific propensity to novelty-seeking [87]. Others authors have not observed any effect on the maturation of new DG cells [64]. These diverging results, summarized in Table 1, may be due differences in experimental designs. One of the main limitations of technical methods is tracking the evolution of new cells *in vivo* studies. Most studies concerning adult neurogenesis use immunohistochemical methods to analyze the proliferation, survival and maturation of newly generated cells in the adult brain. 5-bromo-2-deoxyuridine (BrdU), a synthetic nucleoside and an analogue of thymidine, is the most commonly used marker for detection of newly generated cells. BrdU is incorporated into newly synthesized DNA of dividing cells during the S-phase of the cell cycle. The survival time of animals after BrdU-treatment is determined by whether we are detecting proliferation (short time) or survival (long time) of the BrdU-positive cells. The differentiation and maturation of BrdU-positive cells are determined by the combined use of other specific antibodies. However, the number of exposures, doses and survival time of BrdU vary between studies, which may account for inconsistent data. In drug exposure studies, drugs and BrdU administration are combined, which hinders the comparison of results in relation to the maturation stages of the new cells. In this respect, studies use varying numbers of doses and exposures for each treatment of BrdU and drug, as well as employing different drug administration techniques (passively or self administrated). In addition, BrdU-administration is conducted at different time points, before or after drug treatment, adding more potential variables to the results. Therefore, we must be cautious when interpreting seemingly controversial results, and also when comparing results concerning the same maturation stage.

### 2.2. Effects of Cocaine on the Olfactory Bulb

In relation to the OB, there are few reports of how drugs of abuse can affect this neural system, but it is known that olfactory perception is disrupted in cocaine addicts [91,92]. Current studies demonstrate that administration of stimulant drugs leads to anatomical and chemical changes related to norepinephrines and dopamine neurotransmitters [93-96]. Long-term administration of cocaine reduced the density of norepinephrine reuptake transporter terminals in the OB of rats by approximately one third with respect to the control group. In addition, treated animals showed a decrease in the terminal arbor size [94]. These alterations in the cellular plasticity may have serious consequences for the processing of olfactory information. Although cellular mechanisms underlying the effects of cocaine in the OB are still unclear, recent work has revealed alterations in gene expression in response to acute or chronic treatment. The majority of genes affected by cocaine were up-regulated and encoded proteins involved in membrane trafficking, inhibition of neuronal excitability, cellular energy metabolism, and cellular stress responses [95]. However, this might not be the only cocaine mechanism involved in olfactory perception impairment.

In addition, cocaine disrupts neurogenesis in the SVZ-OB system (summarized in Table 1). Analysis of Ki67 expression, an endogenous marker of cell proliferation, revealed a decrease in proliferation of SVZ after cocaine-treatment in rats [89]. Recently, the number of new cells incorporated into the OB was reported to be reduced in treated animals when the numbers of BrdU positive cells present in the OB were measure a week after administration of the drug [95]. The inhibition of SVZ neurogenesis and, consequently, the interruption of neuroblast migration could lead to relevant functional deficits related with olfactory functions.

## 3. MDMA

MDMA has become a highly popular recreational drug in the last decades and is associated with a range of acute and long-term hazardous effects [97]. MDMA abuse is characterized by alteration of different monoaminergic systems. Principally, it has well documented serotonergic neurotoxic effects that produce a depletion of serotonin axon terminals and decrease serotonin transporter availability [98-102]. Experimental studies have also related MDMA use with increased pro-inflammatory mediators and microglia activation [103-105]. These neurotoxic effects alter a wide range of anatomical regions and cognitive and emotional functions.

### 3.1. Effects of MDMA on the Dentate Gyrus

In relation to the DG and other hippocampus subfields, *in situ* hybridization and immuno-histochemical techniques have shown a down-regulation, and in some cases site-specific, regulation of the expression of different receptors. For example, MDMA administration (20 mg/kg twice daily for four days) caused acute release of both serotonin and corticosteroids with decreased glucocorticoid and mineralocorticoid receptor expression in granule cells of the DG [106]. The receptor expression of serotonin and glucocorticoids exerts an action of sub-regional specificity regulation, which involves differences between the DG and other hippocampus subfields [107]. At the cellular level, the administration of MDMA interferes with mossy fiber activity in the DG [108] and, in combination with alcohol, has been documented to significantly reduce the number of granule cells in the DG and concomitantly increase activated microglia [63].

Studies performed in animal models have shown that MDMA administration also impairs adult neurogenesis (summarized in Table 1). It has been reported that MDMA reduces the proliferation rate under some administration patterns in some cases [109], but not in others [57]. Differences in dosage, duration and route of MDMA and BrdU administration schedules, sex and species used in experimental procedures, may lie behind the different cellular alterations documented. DG proliferation rate is reduced by chronic oral administration of MDMA (1.25 mg/kg-40 mg/kg, for 30 days) in mice. This decrease in the division rate was dose-dependent and affected both sexes. Others authors confirm a proliferative deficit after intensive MDMA treatment (20 mg/kg b.i.d. for 4 d), reporting a 30% reduction of BrdU-positive cells in the DG [110]. In humans, the chronic use of MDMA is not common, but may be applied in a clinical context to treat post-traumatic stress disorder [111]. Binge administration of MDMA best mimics normal human consumption. In such circumstances, MDMA does not reduce the rate of proliferation, but affects cell survival by undermining the survival of new cells in the DG. Administration of MDMA in rats (eight injections of 5 mg/kg at 6 h intervals for two-week intervals of time) has been shown to decrease (ca. 50%) levels of BrdU-labeled cells [57,63].

### 3.2. Effects of MDMA on the Olfactory Bulb

Although MDMA-exposure is related mainly to modifications in serotonin neurotransmitter levels, it also can alter dopamine levels. In this context, the OB is of great relevance, as olfactory cells constitute the major dopaminergic system of the forebrain [112-114]. Moreover, olfactory cells are necessary for processing olfactory information, including discrimination of odorants [15,115]. MDMA-exposure has been documented to increase the release of dopamine in the OB, suggesting that local reinforcing mechanisms may also exist in this brain region [68]. In this regard, MDMA may alter normal function of the OB by modifying dopamine levels.

Besides the neurotoxicity induced by MDMA-administration, this psycho-stimulant alters spontaneous behavior related to psychomotor activity. Thirty minutes after treatment with MDMA, locomotion increases, as does olfactory exploration, which includes sniffing and head movements. However, treatment decreases stimulus-induced behaviors such as jumping, retrieval of food and scratching [116].

In summary, drugs affect neurogenic regions of the adult brain, with the DG being the best documented region in this respect. Cocaine-administration is related with deficits in proliferation of progenitors/precursor cells, whereas MDMA disrupts the survival and maturation of new cell populations. However, other alterations of new adult generated neurons may also be affected. Drugs that induce detrimental effects in adult neurogenesis are dose-dependent, and these effects are reverted after a withdrawal period. Besides adult neurogenesis modifications, exposure to cocaine or MDMA produces a series of alterations in the DG and OB that involve receptors, cellular and synaptic modifications.

## 4. Cocaine- and MDMA-Induced Molecular and Cellular Changes in Neurogenic Systems: Functional Implications

Since the Nottebohm group's studies in canaries [20], it has been proposed that adult neurogenesis may contribute to some types of learning and memory processes [23]. On the other hand, a growing working group has documented a variety of adult neurogenesis-regulating factors that expose the dynamic nature of this process. Inside this variable group of factors, those related to cognitive alterations such as neurodegenerative disease or drug-induced deficits are of particular interest [117-119]. However, the functional contribution of adult neurogenesis to these types of processes is still unknown. To analyze this question in relation to drug-induced impairments, we focus on the changes produced by cocaine and MDMA in the neural systems in which new cells are integrated. These drugs produce a wide range of adult neurogenesis modifications (Table 1).

### 4.1. Drugs Induced Deficits in Neurogenic Systems: Functional Implications

Drugs of abuse are systemically detrimental compounds, but in this review we explore only the neural alterations produced in the functional systems where new neurons are integrated, using MDMA and cocaine as models.

Cocaine and MDMA alter DG synaptic activity. Both acute and chronic administration of cocaine influences DG activity. The amplitude of the LTP induced is decreased in slices from animals that receive 15 mg/kg i.p. of cocaine for two days [80]. It has been documented that repeated administration of cocaine reduces the threshold that must be passed for LTP to be generated, especially in rats that have been sensitized to cocaine, and this correlates with better memory performance in an inhibitory avoidance paradigm [120]. The authors of this study proposed that cocaine can facilitate the acquisition of addictive behavior by influencing the retention of recent memory acquisition. Furthermore, the group of Canales showed that the integrity of the DG is required for a conditioned place preference (CPP) induced by cocaine [43]. This behavior task implies that the retention of associative emotional-contextual memories is similar to the craving behavior induced in drug abusers by context re-exposure. Together, both results suggest that the DG contributes to the retention of persistent drug memories, which characterize addictive behaviors. It has been documented that MDMA also reduces LTP generation and impairs learning in a visual-spatial task [121]. The data obtained shows that DG modifications by cocaine and MDMA depend on the regimen of administration and dose, but in general studies indicate that both drugs alter the synaptic plasticity of the DG. This may underlie the learning deficits and addictive behavior observed in patients who regularly consume drugs. It has been proposed that adult neurogenesis represents a new mechanism of plasticity. The impairments in adult neurogenesis induced by drugs may contribute to LTP impairments and drug behaviors. It has been documented that young neurons produce LTP more readily than mature neurons [122]. This particular property of new cells may be essential to the creation of new memories. This idea is based on the fact that young neurons are more adaptable to new environments and encode new information, while old neurons are more stable and encode old information. In relation to addictive behaviors that make it difficult to forget drug memories, they may be facilitated by a reduction of new cells. Further investigation whether or not adult neurogenesis modification compensates for the synaptic deficits produced by drug exposure. So far it is difficult to link cognitive impairments with a decrease in adult neurogenesis induced by recreational drugs, particularly because of the wide range of neural systems and types of drug-induced molecular modifications.

In this respect, there are few studies that link adult neurogenesis with drug-induced cognitive impairments. One of the most interesting reports to have been published indicates that adult neurogenesis is linked with drug-taking or drug-seeking behaviors [66]. The authors of this study showed that ablation of adult neurogenesis by cranial irradiation increased cocaine self-administration when adult neurogenesis was suppressed before drug-taking, and that it significantly enhanced resistance to extinction of drug-seeking behavior when ablation was carried out after drug-taking. Therefore, these results suggest that adult neurogenesis plays a role in addiction and relapse. The authors proposed that the suppression of new neurons may induce a hippocampal disinhibition, leading to an increased activation of the neural circuit responsible for cocaine-taking. However, this modulation of the function of the hippocampus does not explain the enhanced resistance to drug-seeking behavior. Recent results obtained using cranial irradiation as an adult neurogenesis suppression procedure [123] indicate that a significant decrease in new neurons does not affect the induction of CPP, a process implicated in context-emotional association, suggesting that new neurons are not required to form this type of emotional-context memories. However, previously commented, repeated cocaine administration reduces the threshold of LTP and may induce stronger context-emotional memories. The results suggest that, in some conditions, adult neurogenesis is not required for DG function, specifically in the case of the DG's contribution to context-emotional associations, but that neurogenesis may be more important in the induction of drug seeking behaviors.

Other types of memory that do not have a high emotional association are more difficult to link with adult neurogenesis. It has been described that working memory is impaired by high doses of cocaine self-administration (14 days) in a water maze task (four days after drug treatment) [88]. Once again, the contribution of new cells to the generation of hippocampus LTP and the improvement in encoding of new information are possible mechanisms that facilitate working memory. However, experimental administration of cocaine or MDMA does not produce alterations in a spatial radial maze. Only the combination of MDMA and alcohol-exposure produces long lasting deficits (2 weeks after drug treatment) that affecting working and reference memories, and these deficits are positively correlated with a lower number of granule cells in the DG. Furthermore, MDMA and alcohol administration decrease adult neurogenesis but do not affect learning of spatial tasks [63]. Currently, no causal link has been described between adult neurogenesis and drug-induced impairments. In this review, we have discussed how cocaine and MDMA produce a wide range of anatomical and functional alterations that make it difficult to analyze the contribution of each drug to adult neurogenesis. Furthermore, drug-exposure can induce processes that produce high neurotoxicity in granule cells, which seems to exceed the contribution of adult neurogenesis. For example, the activation of microglia cells and the increase of several pro-inflammatory mediators described in cocaine and MDMA consumption lead to a reduction of functional cells and synaptic activity. The function of adult neurogenesis resides in the understanding of the neural systems in which new cells are fully integrated, and so a comprehensive research approach requires a combination of both fields. In this context, one of the most relevant theories about adult neurogenesis function suggests that new hippocampus cells complement the function of pattern separation of mature granule cells. This hypothesis holds that, without the normal incorporation of new neurons, neurogenic systems are less efficient in determined situations; for example, adult neurogenesis is necessary for the encoded temporal relationship of different stimulus [23]. As occurs with most recreational drugs, cocaine and MDMA impair the normal incorporation of new cells into the DG and OB. If we assume that these systems are necessary for the discrimination of similar events, a less efficient system would make it more difficult to forget memories associated with drugs and/or may underlie impairments of working memory.

### 4.2. Conclusions and Future Perspectives

Neurogenic systems have the capacity to constantly regenerate by creating new neurons during adulthood through a dynamic and highly regulated process known as adult neurogenesis. A wide range of extrinsic and intrinsic factors are reported to reduce adult neurogenesis, and recreational drug use is among them. The mechanisms that have been proposed to interfere with adult neurogenesis are usually classified in two major groups: neurotransmission modifications and increased pro-inflammatory mediators. We have not focused on the mechanism of action of cocaine and MDMA with respect to adult neurogenesis. We have documented how these drugs produce different molecular and cellular changes in neurogenic systems. We believe that that the complexity of these changes, together with a lack of knowledge about neurogenesis system function, make it difficult to evaluate the functional role that new cells play in drug-induced impairments. Current theories about adult neurogenesis function link adult neurogenesis impairment with the impairment of both stable drug memories and working memories that occurs through an undermining of the efficiency of neurogenic systems. Further investigation of the function of adult neurogenic systems is the key to understanding this process.

The function of adult neurogenesis is an issue of great interest, but its full relevance is yet to be elucidated. Before reaching that point, many neural mechanisms need to be understood. Some results indicate that adult neurogenesis is essential in certain situations with increased memory demands [40,124-126] or high emotional interference, such as context conditioning memories [46,47,127] and consolidation of drug-seeking behaviors [89]. It has been reported that adult neurogenesis involves the regulation of affective states in animals in which neurogenesis has been suppressed by genetic manipulation, producing an increase of anxiety-behaviors [128]. Therefore, the role that newly generated neurons play in hippocampus activity and its consequences for anxiety control behaviors may constitute a connection between adult neurogenesis and drug-induced impairments. The modulation of adult neurogenesis by pharmacologic treatments or natural processes may contribute to an increase in the activity of the DG and positively influence anxiety behaviors. Further investigation will no doubt throw light on the contribution of adult neurogenesis to this learning-emotion chain.

## Figures and Tables

**Table 1:** Effects induced by cocaine and MDMA in adult neurogenesis.

Observations	References
• **Cocaine**	
Repeated administration of cocaine induces a decrease in the proliferation rate of DG progenitor cells	[64,65,86]
The acute administration of cocaine does not produce changes in the proliferation rate	[65,80,87]
Impairment of cellular division caused by cocaine consume is normalized after a period of withdrawal	[89]
Chronic cocaine administration may induce an increase of the proliferative rate within the DG after a short-term period of abstinence (2-4 days)	[90]
Chronic cocaine treatment does not affect the survival of new cells	[63,65,87,89]
Effects of cocaine on cell maturation are controversial	[88,87,86,64]
Analysis of Ki67 expression revealed a decrease in proliferation of rat SVZ after cocaine-treatment	[89]
The number of new cells incorporated into the OB a week after BrdU administration was reduced after cocaine-treatment	[95]
	
• **MDMA**	
MDMA reduces the proliferation rate under some administration patterns in some cases, but not in others	[57,109]
The proliferative rate in the DG is reduced after intensive MDMA treatment	[110]
Binge administration of MDMA does not reduce the rate of proliferation, but affects cell survival of new cells in the DG	[57,63]

## Abbreviations

BrdU: 5-bromo-2-deoxyuridine
CPP: conditioned place preference
DG: dentate gyrus
LTD: long term depression
LTP: long term potential
MDMA: *3*,4-Methylenedioxymethamphetamine
NMDA: N-methyl-D-aspartate
NR1/NR2A-2C: recpetors of NMDA
OB: olfactory bulb
PSA-NCAM: polysialylated form of the neural cell adhesion molecule
RMS: rostral migratory stream
SVZ: subventricular zone
